# Anaerobiosis Induced State Transition: A Non Photochemical Reduction of PQ Pool Mediated by NDH in *Arabidopsis thaliana*

**DOI:** 10.1371/journal.pone.0049839

**Published:** 2012-11-21

**Authors:** Sreedhar Nellaepalli, Sireesha Kodru, Malavath Tirupathi, Rajagopal Subramanyam

**Affiliations:** 1 Department of Biochemistry, School of Life Sciences, University of Hyderabad, Hyderabad, India; 2 Department of Plant Sciences, School of Life Sciences, University of Hyderabad, Hyderabad, India; Northwestern University Feinberg School of Medicine, United States of America

## Abstract

**Background:**

Non photochemical reduction of PQ pool and mobilization of LHCII between PSII and PSI are found to be linked under abiotic stress conditions. The interaction of non photochemical reduction of PQ pool and state transitions associated physiological changes are critically important under anaerobic condition in higher plants.

**Methodology/Findings:**

The present study focused on the effect of anaerobiosis on non-photochemical reduction of PQ pool which trigger state II transition in *Arabidopsis thaliana*. Upon exposure to dark-anaerobic condition the shape of the OJIP transient rise is completely altered where as in aerobic treated leaves the rise is unaltered. Rise in *F*_o_ and *F*_J_ was due to the loss of oxidized PQ pool as the PQ pool becomes more reduced. The increase in F_o_′ was due to the non photochemical reduction of PQ pool which activated STN7 kinase and induced LHCII phosphorylation under anaerobic condition. Further, it was observed that the phosphorylated LHCII is migrated and associated with PSI supercomplex increasing its absorption cross-section. Furthermore, evidences from *crr2-2* (NDH mutant) and *pgr5* mutants (deficient in non NDH pathway of cyclic electron transport) have indicated that NDH is responsible for non photochemical reduction of the PQ pool. We propose that dark anaerobic condition accelerates production of reducing equivalents (such as NADPH by various metabolic pathways) which reduce PQ pool and is mediated by NDH leading to state II transition.

**Conclusions/Significance:**

Anaerobic condition triggers non photochemical reduction of PQ pool mediated by NDH complex. The reduced PQ pool activates STN7 kinase leading to state II transition in *A. thaliana*.

## Introduction

The light dependent reactions of photosynthesis involve electron transport through photosystems (PS), PSI and PSII in thylakoid membranes. Upon light absorption by PSII, charge separation occur across the membrane leading to oxidation of water, resulting in electron flow to PSI and ultimately to the reduction of NADP^+^ [1], [2]. The functional and structural coordination between PSI and PSII is required to perform optimal photosynthesis [1], [3].

State transitions is a mechanism of energy redistribution during differential excitation of PSI and PSII that causes changes in redox state of PQ pool in higher plants and green algae under low light conditions [3], [4], [5]. PQ pool and Cyt b_6_/f are the redox carriers which form functional connection between PSII and PSI and establishes the relative absorption cross-section of PSI and PSII [6], [7], [8]. The mechanism of state transitions involves phosphorylation of the light-harvesting chlorophyll (Chl) *a*/*b* proteins (LHCII) by a membrane-bound protein kinase (STN7) which is activated by docking of plastoquinol to the Q_o_ site of Cyt b_6_/f leading to state I to state II transition [9], [10], [11]. Following phosphorylation, a fraction of LHCII migrates to PSI by lateral diffusion; the opposite process occurs after dephosphorylation by phosphatase (PPH1/TAP38) [12], [13], [14]. However, such mechanism of state transitions also occur under conditions irrespective of light due to dark reduction of PQ pool (non photochemical reduction) [15], [16]. This non photochemical reduction could be due to chlororespiration i.e. respiration-like electron transport from NAD(P)H to PQ pool in the thylakoid membranes mediated by NADPH dehydrogenase (NDH) and plastidial terminal oxidase (PTOX) [17], [18], [19]. NDH mediates cyclic electron transport and also participates in electron transfer from stromal reductants to PQ pool indicating its role in chlororespiration. PTOX mediates oxidation of PQ pool by catalyzing electron transfer from reduced PQ pool to ½ O_2_ resulting in formation of H_2_O [19], [20], [21]. Cyclic electron transport which generally takes two routes namely PGR5 mediated electron flow by ferredoxin-quinione oxidoreductase (FQR) and NDH mediated cyclic electron transfer by ferredoxin-NADP reductase (FNR). leads to ΔpH and ATP production [22], [23], [24]. It has been reported that heat stress enhances the dark reduction of PQ pool also indicating the stimulation of cyclic electron transport around PSI [16], [25]. This could be due to higher ATP demand under heat stress resulting in a higher NADPH/ATP ratio favouring non photochemical reduction of PQ pool which in turn provide ATP.

**Figure 1:**
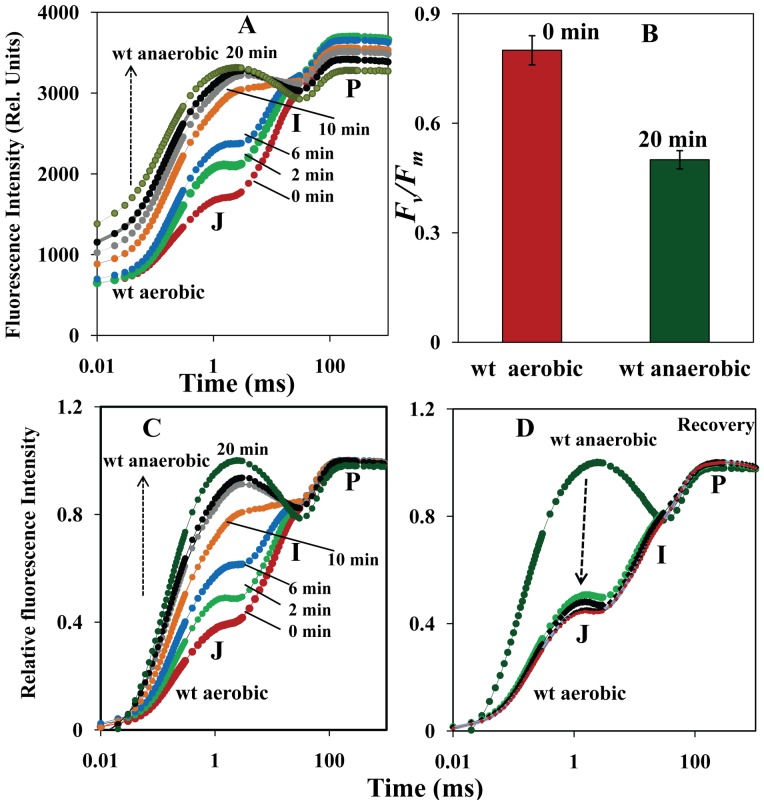
Effect of anaerobiosis on Chl *a* fluorescence transients in *A. thaliana* leaves. A. Raw Chl *a* fluorescence transients of dark anaerobic treated leaves for different time periods (0 to 20 min). B. *F*_v_/*F*_m_ ratio for 0 and 20 min of anaerobic treatment. C. Normalized Chl *a* fluorescence transients of anaerobic treated leaves for different time periods (0 to 20 min). D. Normalized Chl *a* fluorescence transients of recovered leaves to aerobic condition from 20 min of anaerobic condition.

Apart from light, heat and nitrogen deficient conditions anaerobiosis was reported to reduce PQ pool of the photosynthetic electron transport chain [4], [15], [26], [27]. The effect of anaerobiosis on reduction of PQ pool was well studied by OJIP transients [28]. Anaerobic condition results in oxygen-depletion inhibiting the terminal oxidase that generally keeps the PQ-pool in oxidized state ultimately resulting in the reduction of the PQ pool [29]. Similarly, transition to state II was also observed in *A. thaliana* when oxidative phosphorylation is inhibited by inhibitors of respiratory electron transport [30]. This phenomenon was reported to be due to a rapid drop in the ATP content, stimulation of glycolysis and an increase in the NAD(P)H level, which in turn results in non photochemical reduction of the PQ pool [30], [31].

The mechanism of state transitions under anaerobic condition is well documented in *C. reinhardtii*. However, the composition of the photosystems and its light harvesting components are different ranging from cyanobacteria to *C. reinhardtii* and higher plants. This is evident from cyanobacteria where the light harvesting complexes are phycobilisomes [32]. Phosphorylation of LHCII is well studied in *C. reinhardtii* and higher plants under low light conditions. However, the phosphorylation and migration of LHCII differs among these organisms. In higher plants only 15–20% of the LHCII of PSII is transferred to PSI whereas in *C. reinhardtii* ∼80% of the antenna is migrated to PSI upon state II [33], [34], [35], [36]. It has been studied that state I to state II transitions induces a switch from linear to cyclic electron transport. Such changes in electron transport have not been well studied in higher plants. Further, in *C. reinhardtii*, NDH-2 participates in chlororespiration whereas NDH-1 is involved in higher plants chlororespiration [37]. However, the detailed mechanism of PQ reduction and the involvement of NDH and cyclic electron transport under anaerobic condition are still unclear in higher plants.

**Figure 2:**
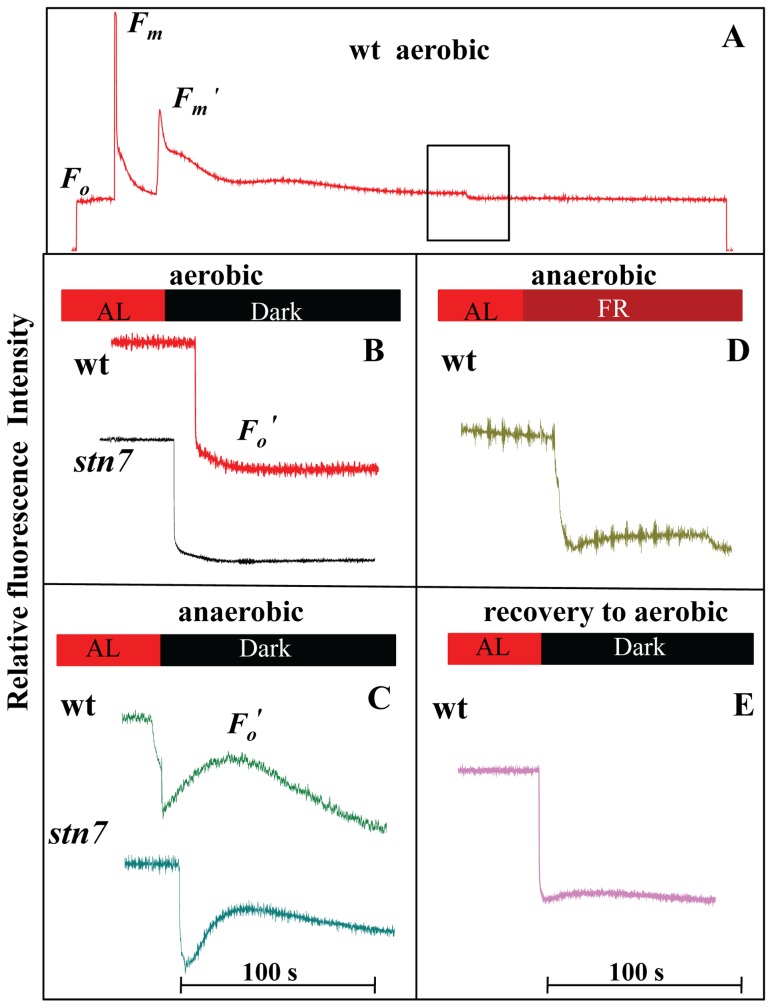
Modulated Chl *a* fluorescence transients. A. Chl *a* fluorescence transient recorded during light to dark transition. B. Post illumination transients of Chl *a* fluorescence in aerobic treated leaves (dark transition) of wt and *stn7*. C. Post illumination transients of Chl *a* fluorescence in anaerobic treated leaves (dark transition) of wt and *stn7*. D. Post illumination transients of Chl *a* fluorescence in anaerobic treated leaves (Far red transition). E. Post illumination transients of Chl *a* fluorescence in recovered leaves to aerobic condition from anaerobic condition (dark transition).

The present study deals with the effect of anaerobiosis on non photochemical reduction of PQ pool and on the mechanism of state transitions in wt *A. thaliana* and mutants *crr2-2* (defective in NdhB subunit of NDH complex) and *pgr5* (defective in PSI CET). Post illumination studies were carried out to monitor changes in non photochemical reduction of PQ pool during anaerobic conditions when compared to aerobic conditions. We investigated the changes in electron transport, phosphorylation pattern of LHCII, and changes in absorption cross-section of PSI and PSII in wt and *stn7*. The importance of NDH in non photochemical reduction of the PQ pool leading to LHCII phosphorylation is also studied under dark anaerobic condition.

**Figure 3:**
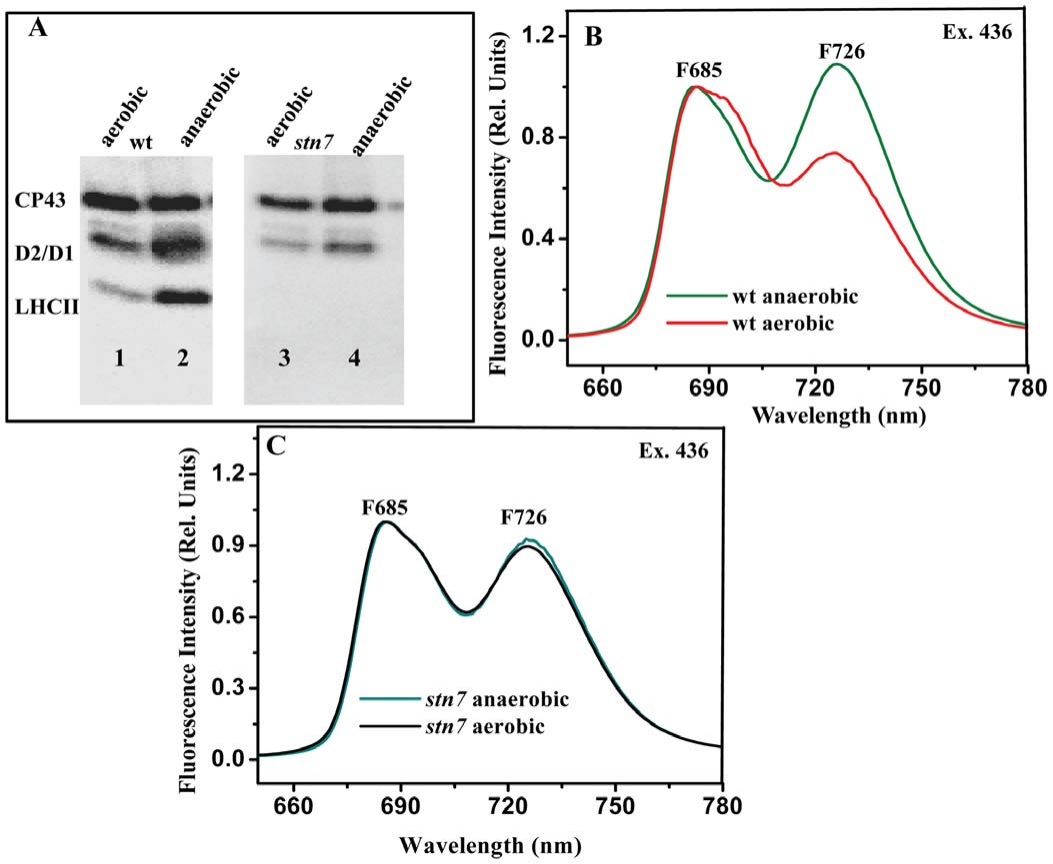
Immunoblotting and 77 K fluorescence analysis. A. Immunoblotting analysis of thylakoid membranes isolated from wt and *stn7,* exposed to 20 min of anaerobic condition. B,C. 77 K fluorescence emission spectra recorded for thylakoid membranes isolated from wt and *stn7,* exposed to 20 min of anaerobic condition.

## Materials and Methods

### Growth Conditions of *Arabidopsis thaliana*

*A. thaliana* wild type (wt), *stn7*, *crr2-2* and *pgr5* mutant plants were grown in controlled environment chambers at 100–120 µmol m^−2^ s^−1^, with 8 h light/16 h dark periods. Leaves were harvested from 6–7 week old plants.

### Anaerobic Treatment

Plants were dark adapted overnight to make the PQ pool oxidized. The leaves were clipped for measuring Chl fluorescence by handy PEA (plant efficiency analyzer) and PAM (pulse amplitude modulator). The detached leaves were placed in the leaf clips of which the sponges were moistened to avoid desiccation during the treatment. These leaf clips were placed in a plastic bag. The head of the measuring equipment (Handy PEA) was also placed inside the bag. To achieve anaerobiosis, N_2_-gas was blown into the bag for 1–20 min; however, some outflow was allowed. The measurements were carried out in N_2_-atmosphere. We note that the Chl *a* fluorescence transients were completely recovered in aerobic condition within 20 min following a 20-min of N_2_-gas treatment. All the experiments were repeated thrice and obtained similar results.

**Figure 4:**
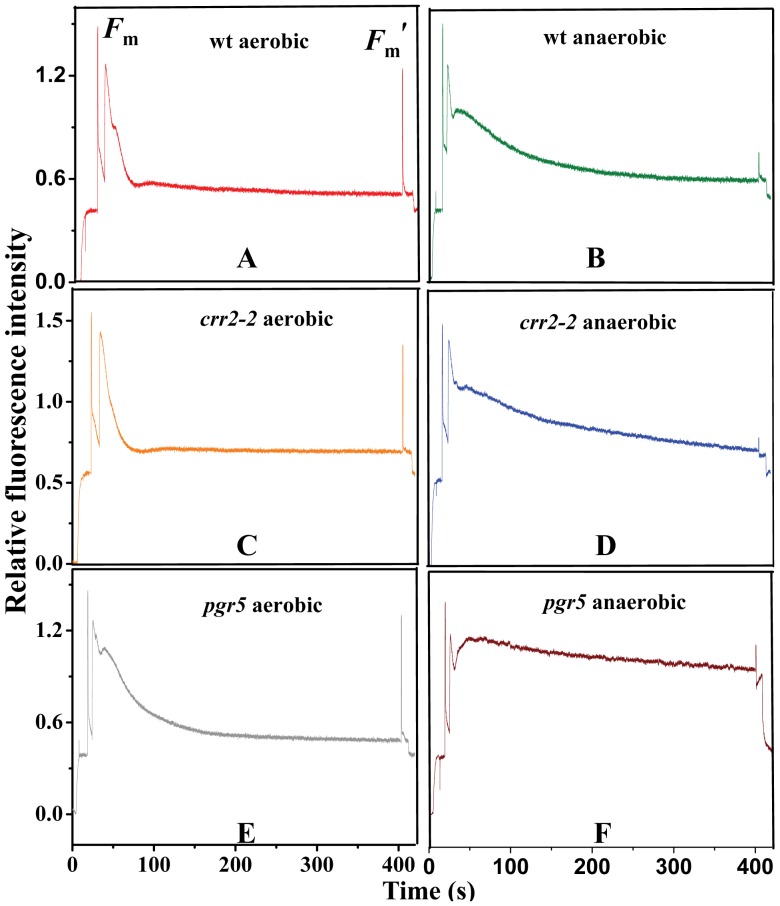
Effect of anaerobic condition under actinic light on Chl fluorescence induction curves. A–C. wt, *crr2-2* and *pgr5* grown under normal conditions, respectively. D–F. wt, *crr2-2* and *pgr5* plants exposed to anaerobic condition, respectively. The leaves were illuminated with actinic light of 35 µmol photons m^−2^ s^−1^ for 8 min and Chl fluorescence were recorded during the anaerobic treatment. The upward and downward arrows indicate on and off of actinic light, respectively. *F*_m_ and *F_m_’* were determined by applying saturating pulse before turning on and before turning off the actinic light, respectively.

### Immunoblotting

For immunoblotting analysis, proteins separated by SDS-PAGE were transferred onto a polyvinyldene difluoride (PVDF) membrane (Bio Rad) using transblot apparatus (Bio Rad), according to manufacturer’s instructions. Blots were probed with anti-phosphothreonine polyclonal antibodies (1/2500 dilution) from New England Biolabs (Cell Signaling Technologies, UK) to detect protein phosphorylation at a threonine residue. Chl concentrations were determined by using Porra et al [38]. Equal Chl concentration (2 µg) was loaded on each lane.

### 77 K Fluorescence Emission Measurements

Isolated thylakoid membranes from different treatments were diluted to10 µg Chl ml^−1^ and excited at 436 nm and emission spectra at 77 K were recorded in the range of 600–780 nm. Low temperature fluorescence emission spectra were measured at 77 K using a (Perkin Elmer, LS-55) fluorescence spectrophotometer. Emission spectra were recorded at a speed of 1 nm s^−1^. Band width was 5 nm for both excitation and emission. Raw spectra were normalized at 685 nm for comparing fluorescence emission bands emanating from PSI.

**Figure 5:**
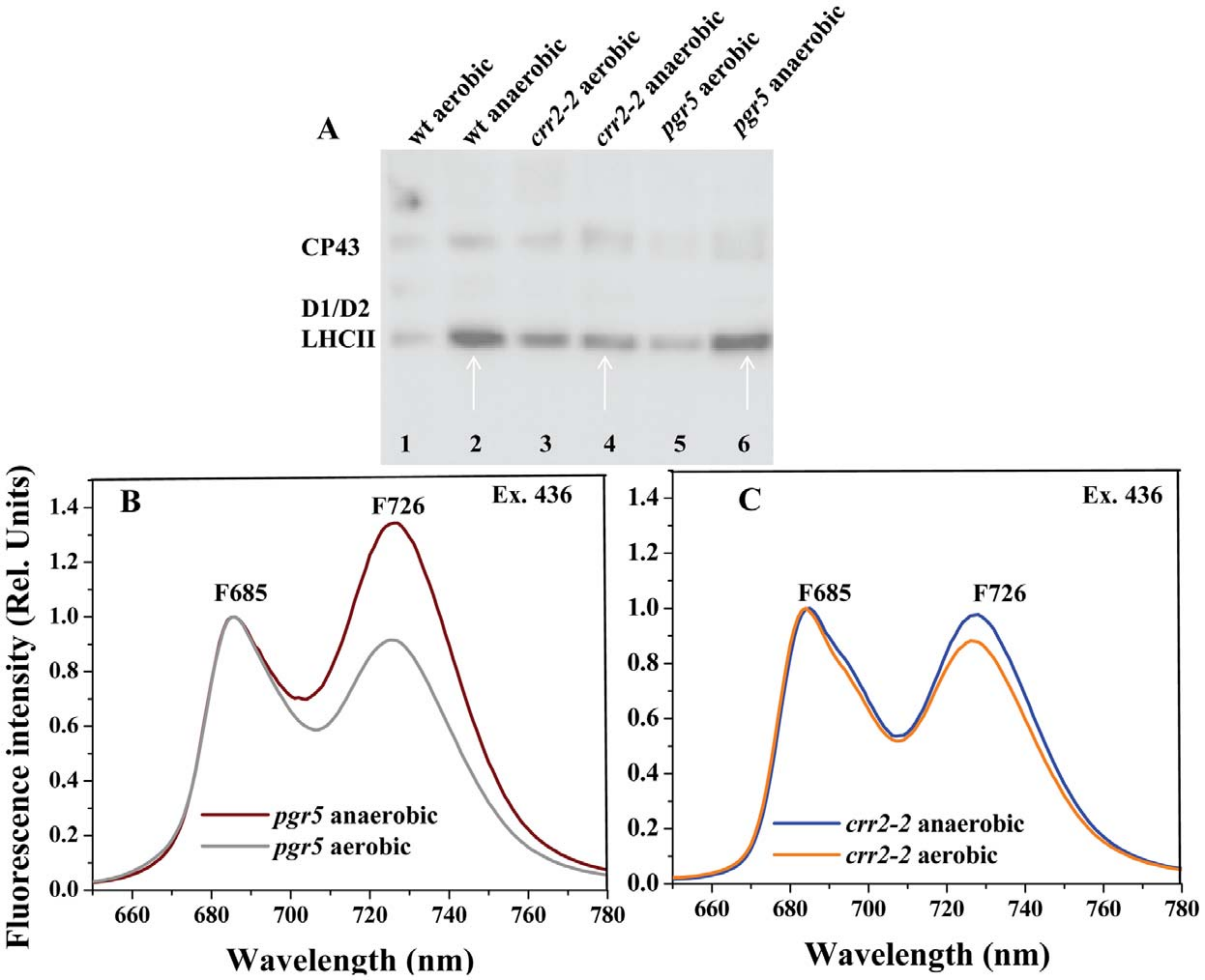
Immunoblotting and 77 K fluorescence emission spectra of thylakoid membranes isolated from wt, *crr2-2* and *pgr5*, exposed to 20 min of anaerobic condition. A. The blots were probed with anti-phosphothreonine. The arrow marks indicated the levels of LHCII phosphorylation in anaerobic treated leaves. B,C. 77 K fluorescence emission spectra recorded for thylakoid membranes isolated from *pgr5* and *crr2-2,* exposed to 20 min of anaerobic condition.

### The Fast OJIP Fluorescence Transient Measurements

Chl fluorescence fast induction curves were measured using Chl fluorimeter (PEA, plant efficiency analyzer, Hansatech, King’s Lynn, Norfolk, UK). Dark adapted leaves were excited by an array of three light-emitting diodes peaking at 650 nm at a photon flux density of 3000 µmol photons m^−2^ s^−1^. The fast fluorescence transients (OJIP) were measured [39] from aerobic and anaerobic treated leaves.

**Figure 6:**
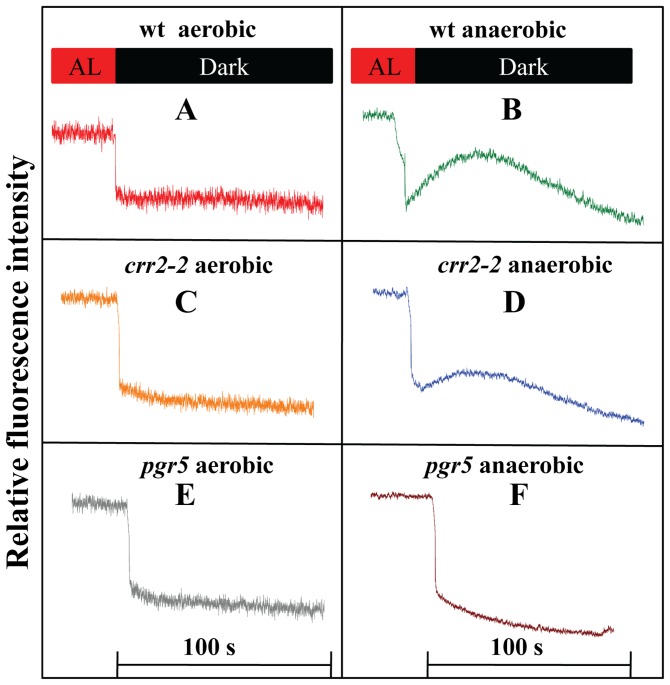
Monitoring non photochemical reduction of PQ pool. A. wt aerobic treated leaves. B. wt anaerobic treated leaves. C. *crr-2-2* aerobic treated leaves. D. *crr-2-2* anaerobic treated leaves. E. *pgr5* aerobic treated leaves. F. *pgr5* anaerobic treated leaves.

### Post Illumination Transients for Monitoring Non Photochemical Reduction of PQ Pool

Chl fluorimeter (dual PAM100, Heinz Walz) was used to monitor Chl *a* fluorescence. Actinic light (AL) illumination was provided by an array of 635-nm LEDs illuminating the surface of the leaf, and applied 1 ms after turning on the 460 nm measuring beam. Post illumination measurements were carried out by applying AL of 35 µmol photons m^−2^ s^−1^ for 8 min and turned off later for 100 s [40]. The maximal fluorescence in the dark-adapted state (*F*_m_) was determined by a 0.8-s saturating (4000 µmol photons m^−2^ s^−1^) light pulse applied after the onset of AL light. The rise in Chl fluorescence in dark is termed as *F*_o_′.

## Results and Discussion

### Effect of Anaerobiosis on Fast Chl *a* Fluorescence Transients

Fast fluorescence transients of Chl *a* reflects the reduction of the electron carriers of PSII and PSI [41], [42]. Reports on Chl fluorescence during anaerobiosis indicated that the PQ pool of the photosynthetic electron transport chain was reduced under anaerobic treatment [31], [43]. Each phase of the fluorescence transient reflects the following: O, minimal Chl *a* fluorescence yield (highest yield of photochemistry) [44]; O-J denotes the reduction of the acceptor of PSII and I-P phase represents the acceptor side of PSII and PSI [45], [46], [47]. J-I phase indicates electron transfer from Q_A_^−^ to Q_B_^−^ and subsequent Q_A_^−^ to Q_B_^2−^ (the reduction of PQ pool) [42], [48]. Since the O-J-I-P fluorescence transient reflects the kinetics and heterogeneity involved in filling up of the redox carriers involved in electron transport from PSII to PSI, consequently, it can be used as a sensitive tool to investigate the photosynthetic apparatus *in vivo* under different environmental conditions [39], [49].

**Figure 7:**
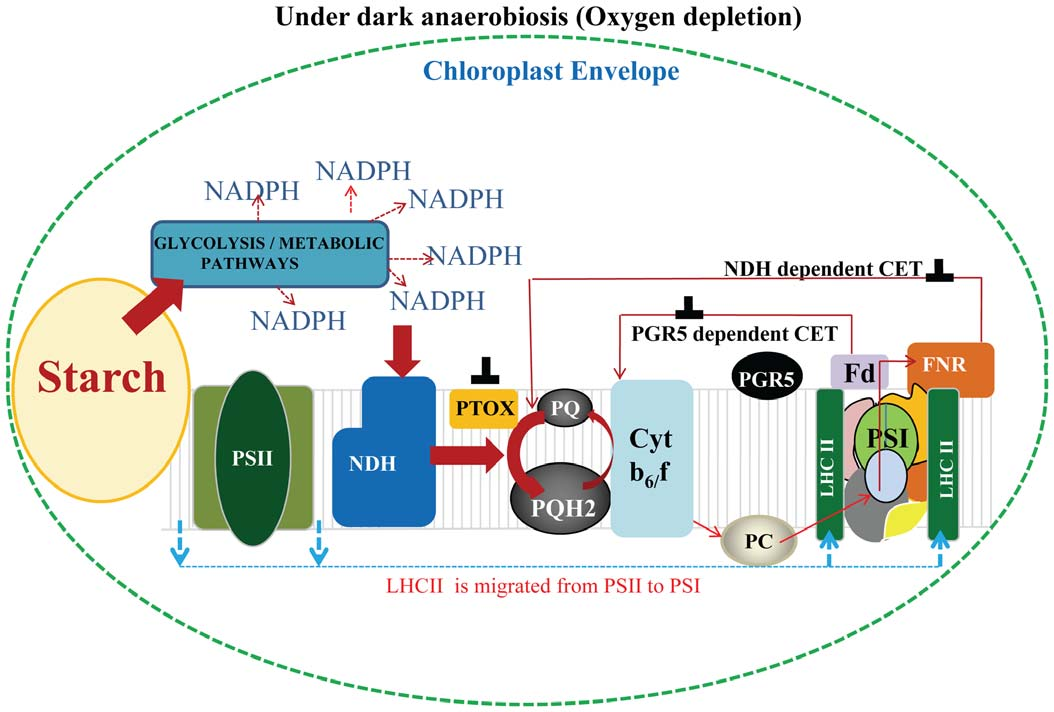
The proposed model of state transitions under anaerobic condition. The non photochemical reduction of the PQ pool under dark anaerobic condition activates STN7 kinase which triggers state II transition (LHCII is migrated from PSII to PSI). During dark condition the acceleration of NDH mediated CET and PGR5 mediated CET was reported to be absent [24]. In the present study, since anaerobic condition was given in dark, the acceleration of CET contributing to the non photochemical reduction could be eliminated. Thus, study with *crr2-2* mutant have shown that NDH mediates the electron transfer from stromal reductants to PQ pool. The accumulation of stromal reductants responsible for dark reduction of the PQ pool could be due to the increased starch breakdown, glycolysis and other metabolic pathways. Anaerobic condition was reported to inhibit mitochondrial respiration thereby blocking the shuttling of redox equivalents between chloroplast and mitochondria. The inverted T corresponds to inhibition of the pathway.

Fig. 1A demonstrates O-J-I-P Chl *a* raw fluorescence transients of anaerobic treated wt leaves from 0 to 20 min. As the duration of anaerobic treatment increased, the rise in the fluorescence transient was significantly altered, increasing minimal fluorescence (F_20 µs_) and decreasing fluorescence yield at P phase (*F_p_*). At 0 min of anaerobic treatment (aerobic condition) there was lower *F*_20 µs_ and showed ∼1, ∼3, and ∼30 ms for J, I, and P phase, respectively. At the end of anaerobic treatment (20 min), the Chl *a* fluorescence transient drastically varied when compared with 0 min of treatment (aerobic condition). The *F*_o_ level increased with the time of exposure to anaerobic treatment while the *F*_p_ level decreased, suggesting a significant decrease in the photochemical yield of *F*_v_/*F*_P_ (in this context) from 0.8 to 0.5 (Fig. 1B). This result was expected because of partial reduction of PQ pool. Also, there has been drastic increase in fluorescence at J phase (F_J_) when the exposure time to anaerobic condition is increased. The rapid increase in J phase might be due to larger reduction of PSII acceptor side and the J step might be the starting point for the over reduction of the PQ pool. This increase in fluorescence yield at 2 ms is usually interpreted as an evidence for accumulation of the reduced Q_A_, possibly due to decreased electron transport beyond Q_A_^−^ [39]. However, the increase in *F*_o_ level by the moderate heat treatment was ascribed to back transfer of electron from the secondary Q_B_^−^ to the stable primary acceptor Q_A_ of PSII [50].

The S_m_, normalized total complementary area above the O-J-I-P transient reflecting multiple turnover of Q_A_ reduction events and N, turn over number: number of Q_A_ reduction events between time 0 and t_Fm_ [42] were drastically reduced as the exposure time increased to anaerobic condition stating that number of multiple turnover Q_A_ reduction events were almost abolished during anaerobic condition (Data not shown). The above fluorescence parameters (S_m_ and N) further indicate that the electron transport has been drastically decreased in anaerobic treated leaves. The area above the OJIP fluorescence transient is generally assumed to be a measure for the number of electrons that have to flow through the electron transport chain in order to reduce the redox carriers of PSII and PSI [46], [51], [52]. In the present study, the reduced area above the fluorescence transient indicates that the electron flow from PSII to PQ pool and PSI is decreased (Fig. 1A,C,D).

Fig. 1C,D are the O-J-I-P transients normalized at the O (*F*_o_) and P (*F*_p_) step in order to see the changes in the transients more clearly. The drastic increase in J phase and drop from J to P phase indicates that reoxidation of PQ was still monitored in anaerobic treated leaves for 20 min. In treatments like DCMU, there was no drop in J to I phase and attains maximal fluorescence at J phase and follows same yield of fluorescence at I and P phase [53]. When the leaves were relaxed to aerobic condition the reappearance of typical OJIP transient was monitored and this suggests that the changes in redox status of the PQ pool are reversible (Fig. 1D). Earlier reports suggested that under anaerobic conditions PTOX was found to be inactivated and the PQ pool was kept in highly reduced state [19], [20], [21]. In this study, chlorophyll fluorimeters are used to measure the redox state of PQ pool (by PEA) and NDH activity (by PAM, post illuminations studies).

### Chl *a* Fluorescence Analysis for Non Photochemical Reduction of PQ Pool

Changes in redox state of PQ pool are further analyzed by post illumination transients (after illuminating with weak actinic light) during aerobic and anaerobic treatment (Fig. 2A–E). Fig. 2A describes Chl *a* fluorescence during light to dark transition. The apparent rise in *F*_o_ (Chl fluorescence increase) after a light to dark transition was used as a measure of non photochemical reduction of the PQ pool and referred as *F*_o_ *′* [54], [55]. Rise in *F*_o_′ level over a period of 100 s after turning off the actinic light was not detectable during dark aerobic treatment indicating the absence of non photochemical reduction of PQ pool in wt and *stn7* (Fig. 2B). Further, upon dark-anaerobic treatment, a rise in *F*_o_′ after switching off the actinic light indicated that non photochemical reduction of the PQ pool was operative in both wt and *stn7* (Fig. 2C). This change in Chl fluorescence level is generally ascribed to the reduction of PQ pool via NDH activity [56], [57] and this activity was not observed under aerobic condition (Fig. 2B,E). Earlier, it has been reported that increase in *F*_o_′ can be ascribed to increased electron transfer from stromal reductants to PQ pool and Cyt b_6_/f mediated by NDH [25]. In higher plants, plastidial NDH complex mediates chlororespiration and cyclic electron transport in thylakoid membranes [19], [26], [56].

When the actinic light was turned off and further illuminating with far-red background which preferentially excites PSI drives the oxidation of PQ pool. When anaerobic treated leaves were illuminated with far red light (electron flow from PSI is accelerated), there is no rise in *F*_o_′ indicating that PSI light oxidized the PQ pool (oxidation of PQ pool by PSI and re-reduction of PQ pool resumed back when switched off the far red light) (Fig. 2D). Anaerobic treated leaves when recovered back to aerobic conditions tend to resume their original state with the PQ pool under oxidized state where non photochemical reduction of the PQ pool was not observed (Fig. 2E). Taken together these observations, it can be suggested that the PQ pool was kept in oxidized state during dark- aerobic conditions, while the PQ pool was under reduced state during dark-anaerobic conditions which is indicative of operation of alternative electron transport mechanisms resulting in non photochemical reduction of PQ pool. Earlier reports suggested that reduction of PQ pool was due to the inability of mitochondrial respiration to dispose metabolically-generated electrons under O_2_ free environment leading to the accumulation of reducing equivalents throughout the cell [58].

### Reduction of PQ Pool Under Anaerobic Condition Triggered State Transition

*stn7* mutant (the expression of the gene responsible for STN7 kinase was blocked) was used to study the role STN7 kinase in phosphorylation of LHCII under anaerobic condition [59]. Since the fluorescence transient rise in *F*_o_ (Fig. 1) and *F*_o_′ (Fig. 2C) is indicative of the redox state of the PQ pool and the redox status of the PQ pool is linked to the LHCII phosphorylation [60], we further aimed to study the changes in LHCII phosphorylation by immunoblotting analysis. Immuno detection with anti phospho-threonine antibodies enabled us to determine changes in phosphorylation levels of PSII proteins especially LHCII. Wt leaves treated under dark aerobic conditions did not exhibit LHCII phosphorylation (Fig. 3A, lane 1). When leaves were exposed to anaerobic condition for 20 min, phosphorylation levels of LHCII were increased drastically (Fig. 3A, lane 2). Dark-anaerobiosis for 20 min would lead to reduction of PQ pool and subsequently activates STN7 kinase which phosphorylates LHCII. STN7 kinase responded to oscillations of the PQ pool redox state in a similar manner as in low light or state II adapted leaves [61]. However, in *stn7* mutant though non photochemical reduction of PQ pool was monitored, LHCII phosphorylation was not observed in both dark aerobic and anaerobic treated leaves (Fig. 3A lane 3 and 4). LHCII phosphorylation was also earlier reported during temperature treatment (in dark), due to non photochemical reduction of PQ pool by stromal reductants [15], [16], [25]. Similarly, Umate et al, (2008) have shown LHCII phosphorylation in anaerobic treated Tobacco leaves [62].

In order to show whether the increase in LHCII phosphorylation is contributing to increase in the PSI absorbance cross-section (phosphorylated LHCII is migrated from PSII to PSI), we further carried out 77 K fluorescence emission analysis. After excitation at 436 nm, PSI gives a significant fluorescence signal peaking at 726 nm, while PSII fluorescence peaks appear mostly at 685 and 695 nm. Fig. 3B shows, the 77 K fluorescence emission spectra of thylakoid membranes isolated from dark aerobic, dark anaerobic adapted wt leaves. For monitoring the changes in PSI (F726), the spectra were normalized at 685 nm which corresponds to PSII emission. In dark, there was no increase in the F726 showing that absorbance cross section of PSI was less due to reduced levels of phosphorylated LHCII (Fig. 3B). In anaerobic condition, the increase in PSI fluorescence emission was due to increased absorption cross-section by binding of LHCII migrated from PSII supercomplex (Fig. 3B). Fluorescence emission at F726 was found to be increased in LL adapted leaves as reported previously [59], [63]. In *stn7*, there is no significant difference between aerobic and anaerobic treated leaves (Fig. 3C) indicating that the phosphorylation of LHCII by STN7 kinase is operational under anaerobic condition in wt. The above results indicate that under anaerobic condition the absorption cross-section of PSI was increased in wt. These results are consistent with the previous reports on anaerobic treatment in *C. reinhardtii* where similar kind of observations were made (LHCII is migrated to PSI under anaerobic conditions which leads to state II) [64].

### Effect of Anaerobiosis on Fluorescence Induction Curves Under Actinic Light Illumination in Mutants Deficient in NDH and PGR5 Mediated Cyclic Electron Transport

The increase in NDH activity was mainly estimated by slow fluorescence rise after actinic light illumination. To investigate the role of chlororespiration and cyclic electron transport under anaerobic conditions, chlororespiratory mutant *crr2-2* (defective in processing of the mRNA of ndhB) and cyclic electron transport mutant *pgr*5 (deficient is defective in the non-NDH pathway of PSI CET) were used for the present study. These mutants were analyzed for Chl *a* fluorescence (Fig. 4) and phosphorylation studies (Fig. 5). Exposure of dark-aerobically treated leaves to actinic light induced initial sharp rise in Chl fluorescence, followed by a small rise in the transient which slowly quenched to a steady-state fluorescence after few min in wt and *crr2-2* (in the presence of light wt could operate NDH dependent cyclic electron transport, whereas *crr2-2* could not operate under anaerobic condition) (Fig. 4A,C). The initial quenching in fluorescence yield in *pgr5* was found to be very slow when compared to wt and *crr-2* (Fig. 4E). In contrast, the Chl *a* fluorescence yield quenching induced by actinic light in anaerobic treated leaves was high in wt, *crr2-2* and *pgr5* (Fig. 4B,D,F). Upon anaerobic treatment wt and *crr2-2* mutant exhibited almost identical transients implying that mutation of NDH dependent cyclic electron transfer did not affect photochemistry under low actinic light.

In *pgr5* mutant, steady state fluorescence (fluorescence quenching was drastically reduced) was observed after few sec of actinic light illumination and this could be due to PSI acceptor side limitation under anaerobic condition (Fig. 4F). The high fluorescence yield in wt and mutants during anaerobic condition could be attributed to the reduction of PQ pool due to inactive PTOX. The increased reduction state of Q_A_ under anaerobic condition likely reflects a high electron pressure on the thylakoid redox system involving PTOX and NDH [19], [56].

### Changes in LHCII Phosphorylation in Mutants *crr2-2* and *pgr5* Upon Anaerobic Treatment

To monitor the changes in LHCII phosphorylation, wt and mutants were subjected to dark aerobic and dark anaerobic treatment. LHCII phosphorylation levels were found to be increased in wt during anaerobiosis confirming that non photochemical reduction of the PQ pool is contributing to the LHCII phosphorylation and leading to a transition to state II. Similarly, when *crr2-2* was subjected to identical conditions as that of wt, LHCII phosphorylation was not observed indicating that NDH is important in dark reduction of PQ pool under anaerobic condition (Fig. 5A). When *pgr5* was subjected to anaerobiosis LHCII phosphorylation was observed. This reduction could be due to the NDH activity of the *pgr5* mutant (Fig. 5A). The increased F726 in *pgr5* is similar to that of wt (Fig. 3B) (state II transition) in anerobically treated leaves, indicating that mutation in PGR5 mediated cyclic electron transport did not affect state II transition (Fig. 5B). However, in *crr2-2* mutant abolition of increase in F726 was monitored showing that NDH is important in dark reduction of PQ pool driving to state transition (Fig. 5C).

To further confirm that NDH is responsible for the non photochemical reduction of the PQ pool during anaerobic conditions, mutants deficient in chlororespiration and cyclic electron transport were analyzed by post illumination transients (Fig. 6). Post illumination transient analysis showed that the wt, *crr2-2* and *pgr5* mutants did not exhibit an increase in *F*_o_′ indicating the absence of NDH activity during dark aerobic condition leading to an oxidized state of PQ pool (state I) (Fig. 6A–F). However, upon anaerobic treatment an increase in *F*_o_′ was monitored for wt indicating enhanced NDH activity (Fig. 6B and Fig. 2B,C). Post illumination transients in *crr2-2* mutant after dark anaerobiosis exhibited a significant decrease in the *F*_o_′ indicating the importance of NDH in non photochemical reduction of the PQ pool (Fig. 6D). Similarly, when *pgr5* mutant was used for the study, the *F*_o_′ rise was completely absent in anaerobic treatment, however state II transition was effectively monitored (Fig. 6F, 5B). This shows the peculiar behavior of *pgr5* under dark anaerobic condition.

In *pgr5*, alternative mechanisms like NDH activity may contribute to the ATP deficiency to a certain extent, but under such abiotic stress conditions promoting higher ATP demand, the impairment of ATP supply might have resulted in significant inhibition of photosynthesis [65], [66]. The contribution of the PGR5-mediated alternative pathway is thus crucial in ATP generation and draining of electrons from PSI under acceptor limiting conditions in O_2_ free air.

### Proposed Mechanism of State Transition Under Anaerobic Condition

Based on our studies we propose that under anaerobic conditions non photochemical reduction of PQ pool mediated by NDH leads to activation of STN7 kinase to phosphorylate LHCII. The phosphorylated LHCII undocks from PSII and migrates to PSI thereby increasing its absorption cross-section and thus leading to state II transition (Fig. 7). The importance of the components CRR2-2 (NdhB) and PGR5 during anaerobic conditions were well documented in the present study where depletion of *crr2-2* affected the non photochemical reduction of PQ pool as well as phosphorylation of LHCII. Under anaerobic conditions reducing equivalents will accumulate in the cell leading to a non photochemical reduction of the PQ pool mediated by NDH activity (Fig. 7). The breakdown of starch and glycolysis and other metabolic processes have been reported to be increased under dark anaerobic condition leading to accumulation of reducing equivalents (NADPH) and further resulted in non photochemical reduction of PQ pool by NDH activity [67].

### Conclusion

The above findings indicate that anaerobiosis in *A. thaliana* would lead to an electron flow which would keep the PQ pool in reduced state. Results with OJIP transients have shown that the drastic increase in J phase under anaerobic condition was due to reduced state of PQ pool. Post illumination transients indicated that the non photochemical reduction of the PQ pool is contributing to the mechanism of state transition where a transition to state II is observed during anaerobiosis in wt and *stn7*. The changes after a transition from dark aerobic to dark anaerobic condition is evident from the LHCII phosphorylation and increase in the PSI absorption cross section. The PQ pool reduction mediated by NDH under anaerobic conditions is confirmed by experiments with *crr2-2* mutant. However, the contribution of the two major pathways (antimycin sensitive as well as antimycin insensitive pathways of cyclic electron transport) which are also involved in the non photochemical reduction of the PQ pool mediated by NDH activity can be ignored under dark anaerobic condition. Though the present study focused on the importance of state transition under anaerobic conditions, the components which are associated with the reduction of PQ pool in presence of light anaerobic condition involving cyclic electron transport are not clear and needs further study.

## References

[1] Nelson N, Ben-Shem A (2004) The complex architecture of oxygenic photosynthesis. Nat Rev Mol Cell Biol 5: 971–82. 15573135 10.1038/nrm1525

[2] Nelson N, Yocum C (2006) Structure and function of photosystems I and II. Annu Rev Plant Biol 57: 521–65. 16669773 10.1146/annurev.arplant.57.032905.105350

[3] Haldrup A, Jensen PE, Henrik CL, Scheller V (2001) Balance of power: a view of the mechanism of photosynthetic state transitions. Trends Plant Sci 6: 301–305. 11435168 10.1016/s1360-1385(01)01953-7

[4] Allen JF (2003) State transitions - a question of balance. Science 299: 1530–1532. 12624254 10.1126/science.1082833

[5] Minagawa J (2011) State transitions; The molecular remodeling of photosynthetic supercomplexes that controls energy flow in the chloroplast. Biochim Biophys Acta 1807: 897–90. 21108925 10.1016/j.bbabio.2010.11.005

[6] Allen AF, Bennett J, Steinback KE, Arntzen CJ (1981) Chloroplast protein phosphorylation couples plastoquinone redox state to distribution of excitation-energy between photosystems. Nature 291: 25–29.

[7] Bennett J, Elizabeth K, Michel H (1988) Cytochrome b6f complex is required for phosphorylation of light-harvesting chlorophyll a/b complex II in chloroplast photosynthetic membranes. Eur J Biochem 171: 95–100. 3338473 10.1111/j.1432-1033.1988.tb13763.x

[8] Zito F, Finazzi G, Delosme R, Nitschke W, Picot D, et al (1999) The Qo site of cytochrome b6f complexes controls the activation of the LHCII kinase. EMBO J 18: 2961–2969. 10357809 10.1093/emboj/18.11.2961 PMC1171378

[9] Allen JF (1992) Protein phosphorylation in regulation of photosynthesis. Biochim Biophys Acta 1098: 275–335. 1310622 10.1016/s0005-2728(09)91014-3

[10] Gal A, Zer H, Ohad I (1997) Redox-controlled thylakoid protein phosphorylation. News and views. Physiol Plant 100: 869–885.

[11] Wollman FA (2001) State transitions reveal the dynamics and flexibility of the photosynthetic apparatus. EMBO J 20: 3623–3630. 11447103 10.1093/emboj/20.14.3623 PMC125556

[12] Bennett J (1980) Chloroplast phosphoproteins. Evidence for a thylakoid-bound phosphoprotein phosphatase. Eur J Biochem 104: 85–89. 6245872 10.1111/j.1432-1033.1980.tb04403.x

[13] Shapiguzova A, Ingelssonb B, Samola I, Andresc C, Kesslerc F, et al (2010) The PPH1 phosphatase is specifically involved in LHCII dephosphorylation and state transitions in *Arabidopsis* . Proc Natl Acad Sci USA 107: 4782–4787. 20176943 10.1073/pnas.0913810107 PMC2842063

[14] Pribil M, Pesaresi P, Hertle A, Barbato RD (2010) Leister. Role of plastid protein phosphatase TAP38 in LHCII dephosphorylation and thylakoid electron flow. PLoS Biol 8 e1000288. 10.1371/journal.pbio.1000288 PMC2811158 20126264

[15] S Nellaepalli, Mekala NR, Zsiros O, Mohanty P, Subramanyam R (2011) Moderate heat stress induces state transitions in *Arabidopsis thaliana* . Biochim Biophys Acta 1807: 1177–1184. 21640068 10.1016/j.bbabio.2011.05.016

[16] Bukhov N, Carpentier R (2004) Alternative photosystem I-driven electron transport routes: mechanisms and functions. Photosynth Res 82: 17–33. 16228610 10.1023/B:PRES.0000040442.59311.72

[17] Johnson GN (2005) Cyclic electron transport in C3 plants: fact or artifact? J Exp Bot 56: 407–416. 15647314 10.1093/jxb/eri106

[18] Shikanai T, Endo T, Hashimoto T, Yamada Y, Asada K, et al (1998) Directed disruption of the tobacco ndhB gene impairs cyclic electron flow around photosystem I. Proc Natl Acad Sci USA. 95: 9705–9709. 10.1073/pnas.95.16.9705 PMC21403 9689145

[19] Peltier G, Cournac L (2002) Chlororespiration. Ann Rev Plant Biol 53: 523–550. 12227339 10.1146/annurev.arplant.53.100301.135242

[20] Joet T, Cournac L, Peltier G, Havaux M (2002a) Cyclic electron flow around photosystem I in C-3 plants. In vivo control by the redox state of chloroplasts and involvement of the NADH dehydrogenase complex. Plant Physiol 128: 760–769. 11842179 10.1104/pp.010775 PMC148937

[21] Joet T, Genty B, Josse EM, Kuntz M, Cournac L, et al (2002b) Involvement of a plastid terminal oxidase in plastoquinone oxidation as evidenced by expression of the *Arabidopsis thaliana* enzyme in tobacco. J Biol Chem 277: 31623–31630. 12050159 10.1074/jbc.M203538200

[22] Munekage Y, Hojo M, Meurer J, Endo T, Tasaka M, et al (2002) PGR5 is involved in cyclic electron flow around photosystem I and is essential for photoprotection in *Arabidopsis* . Cell 110: 361–371. 12176323 10.1016/s0092-8674(02)00867-x

[23] Endo T, Shikanai T, Sato F, Asada K (1998) NAD(P)H dehydrogenase-dependent, antimycin A-sensitive electron donation to plastoquinone in tobacco chloroplasts. Plant Cell Physiol 39: 1226–1231.

[24] Munekage Y, Shikanai T (2005) Cyclic electron transport through photosystem I. Plant Biotechnol. 22: 361–369.

[25] Havaux M (1996) Short-term responses of photosystem I to heat stress. Induction of a PS II-independent electron transport through PS I fed by stromal components. Photosynth Res 47: 85–97. 24301710 10.1007/BF00017756

[26] Peltier G, Schmidt GW (1991) Chlororespiration: An adaptation to nitrogen deficiency in *Chlamydomonas reinhardtii* (photosynthesis/chloroplast/thylakoid/NADH-plastoquinone oxidoreductase/cytochrome. Proc Natl Acad Sci USA 88: 4791–4795. 11607187 10.1073/pnas.88.11.4791 PMC51752

[27] Garab G, Lajko F, Mustardy L, Marton L (1989) Respiratory control over photosynthetic electron transport in chloroplasts of higher plant cells. Evidence for chlororespiration. Planta 179: 349–358. 24201664 10.1007/BF00391080

[28] Schreiber U, Vidaver W (1974) Chlorophyll fluorescence induction in anaerobic *Scenedesmus obliquus* . Biochim Biophys Acta 368: 97–112. 4423963 10.1016/0005-2728(74)90100-5

[29] Cournac L, Redding K, Ravenel J, Rumeau D, Josse E-M, et al (2000) Electron flow between photosystem II and oxygen in chloroplasts of photosystem I-deficient algae is mediated by a quinol oxidase involved in chlororespiration. J Biol Chem 275: 17256–17262. 10748104 10.1074/jbc.M908732199

[30] Rebeille F, Gans P (1988) Interaction between chloroplasts and mitochondria in microalgae: role of glycolysis. Plant Physiol 88: 973–975. 16666488 10.1104/pp.88.4.973 PMC1055696

[31] Bennoun P (1994) Chlororespiration revisited: mitochondrial plastid interactions in *Chlamydomonas* . Biochim Biophys Acta 1186: 59–66.

[32] Piven I, Ajlani G, Sokolenko A (2005) Phycobilisome linker proteins are phosphorylated in *Synechocystis* sp. PCC 6803. J Biol Chem 280: 21667–21672. 15805115 10.1074/jbc.M412967200

[33] Delosme R, Olive J, Wollman FA (1996) Changes in light energy distribution upon state transitions: an *in vivo* photoacoustic study of the wild type and photosynthesis mutants from *Chlamydomonas reinhardtii* . Biochim Biophys Acta 1273: 150–158.

[34] Finazzi G, Rappaport F, Furia A, Fleischmann M, Rochaix J-D, et al (2002) Involvement of state transitions in the switch between linear and cyclic electron flow *Chlamydomonas reinhardtii* . EMBO Reports 3: 280–285. 11850400 10.1093/embo-reports/kvf047 PMC1084013

[35] Stauber EJ, Fink A, Markert C, Kruse O, Johanningmeier U, et al (2003) Proteomics of *Chlamydomonas reinhardtii* light-harvesting proteins. Eukaryotic Cell 2: 978–994. 14555480 10.1128/EC.2.5.978-994.2003 PMC219354

[36] Mozzo M, Mantelli M, Passarini F, Caffarri S, Croce R, et al (2010) Functional analysis of Photosystem I light-harvesting complexes (Lhca) gene products of *Chlamydomonas reinhardtii.* . Biochim Biophys Acta 1797: 212–221. 19853576 10.1016/j.bbabio.2009.10.005

[37] Mus F, Cournac L, Cardettini V, Caruana A, Peltier G (2005) Inhibitor studies on nonphotochemical plastoquinone reduction and H_2_ photoproduction in *Chlamydomonas reinhardtii* . Biochim Biophys Acta 1708: 322–32. 15950924 10.1016/j.bbabio.2005.05.003

[38] Porra RJ, Thompson WA, Kriedemann PE (1989) Determination of accurate extinction coefficients and simultaneous equations for assaying chlorophylls *a* and *b* extracted with four different solvents: Verification of the concentration of chlorophyll standards by atomic absorption spectroscopy. Biochim Biophys Acta 975: 384–394.

[39] Strasser RJ, Srivastava A (1995) Govindjee (1995) Polyphasic chlorophyll *a* fluorescence transients in plants and cyanobacteria. Photochem Photobiol 6: 32–42.

[40] Kovacs L, Damkjaer J, Kereiche S, Ilioaia C, Ruban AV, et al (2006) Lack of the light-harvesting complex CP24 affects the structure and sunction of the grana membranes of higher plant chloroplasts. Plant Cell 18: 3106–3120. 17114352 10.1105/tpc.106.045641 PMC1693946

[41] Strasser RJ, Tsimilli-Michael M, Srivastava A (2004) Analysis of the chlorophyll *a* fluorescence transient. In: Papageorgiou GC, Govindjee (eds) Chlorophyll fluorescence: a signature of photosynthesis. Advances in photosynthesis and respiration vol 19. Springer, Dordrecht, The Netherlands, 321–362.

[42] Stirbet A (2011) Govindjee (2011) On the relation between the Kautsky effect (chlorophyll *a* fluorescence induction) and photosystem II: basics and applications of the OJIP fluorescence transient. J Photochem Photobiol B Biol 104: 236–257. 10.1016/j.jphotobiol.2010.12.010 21295993

[43] Tóth SZ, Schansker G, Strasser RJ (2007) A non-invasive assay of the plastoquinone pool redox state based on the OJIP-transient. Photosynth Res 93: 193–203. 17487568 10.1007/s11120-007-9179-8

[44] Reto Strasser J, Govindjee (1991) The Fo and the O-J-I-P Fluorescence Rise in Higher Plants and Algae. Regulation of Chloroplast Biogensis. (Ed. J.H. Argyroudi-Akoyunoglou) Plenum Press, New York. pp, 423–426.

[45] Ilik P, Schansker G, Kotabova E, Vaczi P, Strasser RJ, et al (2006) A dip in the chlorophyll fluorescence induction at 0.2–2 s in Trebouxia-possesing lichens reflects a fast reoxidation of photosystem I. A comparison with higher plants. Biochim Biophys Acta 1757: 12–20. 16403432 10.1016/j.bbabio.2005.11.008

[46] Schansker G, Toth SZ, Strasser RJ (2006) Dark-recovery of the Chl *a* fluorescence transient (OJIP) after light adaptation: the qT-component of nonphotochemical quenching is related to an activated photosystem I acceptor side. Biochim Biophys Acta 1757: 787–797. 16777056 10.1016/j.bbabio.2006.04.019

[47] Ceppi MG, Oukarroum A, Cicek N, Strasser RJ, Schansker G (2012) The IP amplitude of the fluorescence rise OJIP is sensitive to changes in the photosystem I content of leaves: a study on plants exposed to magnesium and sulfate deficiencies, drought stress and salt stress. Physiol Plantarum 144: 277–288. 10.1111/j.1399-3054.2011.01549.x 22121914

[48] Govindjee (2004) Chlorophyll *a* fluorescence: a bit of basics and history, in: G.C. Papageorgiou, Govindjee (Eds.), Chlorophyll *a* Fluorescence: A Signature of Photosynthesis, Advances in Photosynthesis and Respiration, vol. 19, Springer, Dordrecht, The Netherlands, 1–41.

[49] Joly D, Carpentier R (2009) Sigmoidal reduction kinetics of the photosystem II acceptor side in intact photosynthetic materials during fluorescence induction. Photochem Photobiol Sci 8: 167–173. 19247508 10.1039/b815070b

[50] Ducruet M (1999) Relation between the heat induced increase of Fo fluorescence and a shift in the electronic equilibrium at the acceptor side of PSII. Photosynthetica 37: 335–338.

[51] Joliot P, Joliot A (2002) Cyclic electron transport in plant leaf. Proc. Natl. Acad. Sci. USA 99: 10209–10214. 10.1073/pnas.102306999 PMC126649 12119384

[52] Strasser BJ, Strasser RJ (1995) Measuring fast fluorescence transients to address environmental questions: the JIP-test. In: Mathis P, ed. Photosynthesis: from light to biosphere, Dordrecht: Kluwer Academic Publishers. 977–980.

[53] Tóth SZ, Schansker G, Strasser RJ (2005) In intact leaves, the maximum fluorescence level (F(M)) is independent of the redox state of the plastoquinone pool: a DCMU-inhibition study. Biochim Biophys Acta 1708: 275–82. 15869738 10.1016/j.bbabio.2005.03.012

[54] Mano J, Miyake C, Schreiber U, Asada K (1995) Photoactivation of the electron flow from NADPH to plastoquinone in spinach chloroplasts. Plant Cell Physiol 36: 1589–1598.

[55] Farineau J (1999) Study of the non-photochemical dark rise in chlorophyll fluorescence in pre-illuminated leaves of various C3 and C4 plants submitted to partial anaerobiosis. Plant Physiol Biochem 37: 911–918.

[56] Rumeau D, Peltier G, Cournac L (2007) Chlororespiration and cyclic electron flow around PSI during photosynthesis and plant stress response. Plant Cell Environ 30: 1041–1051. 17661746 10.1111/j.1365-3040.2007.01675.x

[57] Burrows PA, Sazanov LA, Svab Z, Maliga P, Nixon PJ (1998) Identification of a functional respiratory complex in chloroplasts through analysis of tobacco mutants containing disrupted plastid *ndh* genes. EMBO J 17: 868–876. 9463365 10.1093/emboj/17.4.868 PMC1170436

[58] Hoefnagel MHN, Atkin OK, Wiskich JT (1998) Interdependence between chloroplasts and mitochondria in the light and the dark. Biochim Biophys Acta 1366: 235–255.

[59] Bellafiore S, Barneche F, Peltier G, Rochaix JD (2005) State transitions and light adaptation require chloroplast thylakoid protein kinase STN7. Nature 7028: 892–5. 10.1038/nature03286 15729347

[60] Vener AV, van Kan PJ, Rich PR, Ohad I, Andersson B (1997) Plastoquinol at the quinol oxidation site of reduced cytochrome bf mediates signal transduction between light and protein phosphorylation: thylakoid protein kinase deactivation by a single-turnover flash. Proc Natl Acad Sci USA 94: 1585–1590. 11038603 10.1073/pnas.94.4.1585 PMC19835

[61] Tikkanen M, Grieco M, Aro EM (2011) Novel insights into plant light-harvesting complex II phosphorylation and ‘state transitions’. Trends Plant Sci 16: 126–131. 21183394 10.1016/j.tplants.2010.11.006

[62] Umate P, Fellerer C, Schwenkert S, Zoryan M, Eichacker LA, et al (2008) Impact of PsbTc on forward and back electron flow, assembly, and phosphorylation patterns of photosystem II in Tobacco. Plant Physiol 148: 1342–1353. 18805952 10.1104/pp.108.126060 PMC2577276

[63] Kouril R, Zygadlo A, Arteni AA, de Wit CD, Dekker JP, et al (2005) Structural characterization of a complex of Photosystem I and light harvesting complex II of *Arabidopsis thaliana* . Biochemistry 44: 10935–10940. 16101276 10.1021/bi051097a

[64] Subramanyam R, Jolley C, Brune DC, Fromme P, Webber AN (2006) Characterization of a novel Photosystem I–LHCI supercomplex isolated from *Chlamydomonas reinhardtii* under anaerobic (State II) conditions. FEBS Lett 580: 233–238. 16375899 10.1016/j.febslet.2005.12.003

[65] Munekage YN, Genty B, Peltier G (2008) PGR5 Impairment on Photosynthesis and Growth in *Arabidopsis thaliana* . Plant Cell Physiol 49: 1688–1698. 18799484 10.1093/pcp/pcn140

[66] Shikanai T (2007) Cyclic electron transport around photosystem I: Genetic approaches. Annu Rev Plant Biol 58: 199–217. 17201689 10.1146/annurev.arplant.58.091406.110525

[67] Johnson X, Alric J (2012) Interaction between starch breakdown, acetate assimilation and photosynthetic cyclic electron flow in *Chlamydomonas reinhardtii* . J Biol Chem 287: 26445–52. 22692199 10.1074/jbc.M112.370205 PMC3406727

